# Building an evaluation model for the motor health of preschool-aged children from the perspective of interdisciplinary integration

**DOI:** 10.3389/fpsyg.2025.1580843

**Published:** 2025-06-16

**Authors:** Dongxu Du, Linyan Chai, Gao Yang, Xiang Ao

**Affiliations:** ^1^Sports Academy, Zunyi Normal University, Zunyi, China; ^2^Surgical Center for Neurological Diseases, Zunyi First People’s Hospital, Zunyi, China; ^3^Department of Physical Education, Guiyang Preschool Education College, Guiyang, China; ^4^College of Sports Science, Guangxi College for Preschool Education, Guangxi, China

**Keywords:** motor health, preschool children, evaluation model, interdisciplinary, mental health

## Abstract

**Introduction:**

This study explored a structural equation model of preschool children’s sports health levels and revealed the coupling effect of many factors on children’s sports health levels. Despite the importance of motor health in early childhood, a comprehensive evaluation model integrating multiple health dimensions remains underexplored.

**Methods:**

WPS Office 6.4 and IBM^®^ SPSS^®^ Statistics 26.0 were used to analyze the data, and the statistical significance level was set at *p*-value < 0.05. IBM^®^ SPSS^®^ Amos™ 24.0 was used to analyze the data and develop a structural equation model for children’s motor health.

**Results:**

The total motor health scores of the preschoolers showed highly significant positive correlations (*p*-value < 0.01) at the 0.01 level (two-tailed) with motor ability, physical health, mental health, social and emotional competence, motor health environment, and motor health behavior. Through the evaluation and verification of the fitness of the whole and multi-group structural equation models, it can be seen that the significance probability values of the preset theoretical model are *p*-value > 0.05, RMSEA < 0.05, GFI > 0.90, and AGFI > 0.90, which supports the hypothesis of nothingness. NFI > 0.90, IFI > 0.90, TLI > 0.90, and CFI > 0.90. All indices of value-added fitness met the acceptable standards of the model, and the model met the standard of simple adaptation.

**Conclusion:**

A structural equation model of preschool children’s motor health was constructed, which passed the fitness test and is stable, adaptable, and widely applicable. These findings can help educators design evidence-based physical activity programs to enhance preschoolers’ motor and overall health.

## 1 Introduction

Early childhood development refers to the cognitive, physical, language, motor, social, and emotional development of children from 0 to 8 years of age (World Health Organization, 2020). Health promotion should be implemented at all ages because it may reduce the risk of, or prevent disease (Razeghi et al., 2020). Activities for preschoolers interacting with parents or caregivers that do not involve screens as a form of entertainment are beneficial to children’s health. Examples include reading, singing, storytelling, coloring, building blocks, cutting paper, and puzzles or games (World Health Organization, 2019). Interactive play with parents or caregivers promotes children’s cognitive and learning and motor skills (World Health Organization, 2018). Preschool children’s health is a pressing issue with respect to obesity, cardiovascular disease, metabolic syndrome, and type 2 diabetes. Exercise is a key factor in healthy child development that mitigates childhood obesity, and promotes metabolic health.

The behavior of individuals is closely influenced by the environment in which it occurs (Yao et al., 2021). Children of preschool age have the highest rate of learning and cognitive development, and exercise is beneficial for the development of their physical and mental health (Jia et al., 2021). Motor skills development and physical education classes have a significant impact on preschoolers’ physical and mental health and learning abilities (Wang et al., 2023). Social-emotional competence (promoted from ages 3–6) results in positive behavioral outcomes and is a key factor in academic success (Martinsone et al., 2022). Promoting physical activity in preschoolers is a critical intervention to optimize health and prevent chronic disease. Establishing and maintaining a healthy and active lifestyle among preschoolers is critical, with parents being the most powerful role models for controlling young children’s behaviors, diets, and activity environments (Goldfield et al., 2012). Shaping and promoting positive behaviors early in life is critical to developing lifelong healthy habits, and a child’s survival environment has a powerful influence on behavior (Adamo et al., 2014). Participation in physical activity plays an important role in promoting physical and mental health; however, physical activity among preschoolers in Shanghai, China, is inadequate (Quan et al., 2019).

Physical and mental health appear to play crucial roles in children’s normal development (Cocca et al., 2020). A mental health status recognition model for physical activity has been established, demonstrating significant enhancement effects of exercise on mental health (Li et al., 2020). A relationship model between physical activity and physical-mental health was constructed, explaining 35.8% of the variance in mental health (Congsheng et al., 2022). Cultural differences in physical education curriculum and sports participation may influence disparities in children’s motor competence and health development (Luz et al., 2022). Physical activity and motor competence are recognized as closely interrelated factors in preventing childhood obesity, with motor competence showing correlations at individual, familial, and environmental levels. The socio-ecological determinants of children’s mental health are multidimensional, with individual-related factors emerging as the most significant predictors of mental health (Niemistö et al., 2020). A positive correlation exists between nutrition-exercise behaviors and health literacy levels (Ayaz-Alkaya and Kulakçı-Altıntaş, 2021). Preschoolers’ health-related quality of life increases with adherence to WHO guidelines for physical activity, sedentary behavior, and sleep duration (Chia et al., 2020). Physical activity demonstrates potential for improving mental health and psychological states (Okuyama et al., 2021). Motor competence appears to exert positive effects on children’s health indicators (Pombo et al., 2023). A multilevel bioecological analysis of factors influencing children’s mental health and psychological well-being emphasizes the critical importance of environmental and social factors in shaping children’s mental health (Arakelyan and Ager, 2020). Current research reveals abundant findings on influencing factors of preschool children’s physical activity health, yet existing evaluation models exhibit deficiencies in theoretical frameworks, methodological limitations, and practical implementation challenges. Therefore, this study proposes a novel interdisciplinary approach integrating Bronfenbrenner’s ecological systems theory, Bandura’s social learning theory, CASEL’s social-emotional learning framework, comprehensive evaluation methodologies, exercise science, and psychological theories to construct a preschool children’s physical activity health assessment model with enhanced theoretical and practical value.

Evaluation science has also developed along with decision science. The evaluation process involves judging observations according to certain criteria and assigning meanings and values to them. Observations can only reflect the current situation, whereas evaluations can judge their meanings. Structural equation modeling (SEM), which some scholars also call latent variable modeling (Moustaki et al., 2004). SEM is also known as linear structural relationship modeling, covariance structural analysis, latent variable analysis, validation factor analysis, and simple LISREL analysis (Hair, 2010).

Usually, SEM is categorized as higher statistics, which belongs to multivariate statistics, and integrates factor and path analysis, to test the relationship between the dominant, latent, and interfering or error variables in the model, and thus to obtain the direct, indirect, or total effect of the independent variable on the dependent variables. The image buttons in the depiction toolbox can be used to quickly draw SEM graphs, browse estimated model maps, modify model maps, evaluate model fit and reference correction metrics, and output the best model (Wu, 2010).

This study uses the key indicators of motor ability, physical health, mental health, motor health behavior, social-emotional ability, and motor health environment, according to their intrinsic connections, closely combined with the actual work of motor health, processing, and refining, to evaluate the level of motor health in preschool children. We adopted statistical models, constructed hypothetical model diagrams, selected the best-fitting model based on evaluation criteria and corrected indicators, and provided insights to inform motor health practices.

In this study, we explored the internal contradiction between the ecological holism of preschool children’s sports health evaluation and realistic traditional parenting education, as well as the external contradiction between the idealized overall evaluation of the ecosystem and environmental reality. In addition, we used SEM to explore preschool children’s sports health levels and revealed the coupling effect of many factors on children’s sports health levels. The validation and innovation of the model will help promote the in-depth development of research in this field, providing a theoretical reference for the evaluation of children’s sports health and promoting its coordinated development.

The innovative contents of this article are as follows: in view of the challenges faced by children’s development in post-modern society, it is of great significance to build a preschool children’s sports health model based on an interdisciplinary approach. The global perspective advocates constantly transcending the limitations of human health and wellbeing and promoting children’s health as an important part of the whole of mankind.

## 2 Materials and methods

### 2.1 Participants

The sample size for a stable and fit SEM is between 200 and 500 (Stegmann, 2017). Therefore, a random whole cluster sampling method was used to select 410 preschool children, including 201 boys and 209 girls, from three districts in Guizhou Province, Southwest China, as the study population. From December 2023 to February 2024, the research team organized motor ability assessments of preschool children and a questionnaire survey for parents in two kindergartens in the county’s urban area and three kindergartens in the municipal area. Data collection was strictly conducted in compliance with ethical research requirements, with informed consent and signed documentation obtained from administrators of relevant Chinese kindergartens, volunteer parents, and participating children. This process rigorously ensured children’s voluntary participation and implemented comprehensive safety protocols. This study was approved by the Ethics Committee of Mahasarakham University (#497-453/2023).

### 2.2 Selection criteria and screening

The inclusion criteria were as follows: (1) the children showed normal intellectual development and no organic disease; (2) they had completed physical fitness tests using the National Physical Fitness Test Standards (2023) (early childhood section); and (3) children’s families were informed about the study, voluntarily participated, and signed the consent form.

The exclusion criteria were as follows: (1) the children and parents refused or did not cooperate with the test and questionnaire; (2) the children were suffering from systemic infectious diseases; (3) had congenital diseases such as severe heart, liver, and kidney insufficiency and heart disease; and (4) had been diagnosed with cerebral palsy, intellectual disability, autism spectrum disorder, attention deficit hyperactivity disorder, or other diseases.

### 2.3 Data extraction

Data was gathered from December 2023 to February 2024. In the first data collection phase, preschool children were assessed on body shape (height and weight) and motor ability (grip strength, standing long jump, sitting forward bend, two-legged jump, 15-m run around obstacles, and walking on a balance beam). In the second phase, data on motor health level was collected from the preschool children who had been tested for motor ability and selected as the research subjects, with the cooperation and help of the kindergarten staff. The parents signed the informed consent form, and the parental proxy questionnaire was distributed through WeChat^®^. Data on the children’s physical and mental health were collected using the German version of the 3–6 years old quality of life questionnaire (KINDLR) represented by parents. The social and emotional learning scale represented by parents was used to collect data on the children’s social and emotional abilities; the sports health environment (MHES) scale represented by parents was used to collect data on the children’s sports health environment; and the sports health behavior (MHBS) scale represented by parents was used to collect data on the children’s sports health behavior.

### 2.4 Data analysis

WPS Office 6.4 and IBM^®^ SPSS^®^ Statistics 26.0 were used to analyze the data, with the significance set at *p*-value < 0.05. IBM^®^ SPSS^®^ Amos™ 24.0 was used for statistical analysis and to develop an SEM for children’s motor health. SEM is an important statistical method for quantitative research in contemporary behavioral and social fields. The measurement model consists of latent variables, which are derived from measurement instruments such as scales or questionnaires, and observed variables, which are traits or abstract concepts formed among the observed variables that cannot be measured directly but are inferred from measured data. The analysis procedure for the model has eight steps: (1) conceptualization of the model, (2) construction of the path diagram, (3) confirmation of the model, (4) identification of the model, (5) estimation of the parameters, (6) evaluation of the model fit, (7) modification of the model, and (8) review and calibration of the model. There are three main methods for evaluating structural equation model fit: (i) absolute fitness statistics, including CMIN/DF, RMR, SMRM, RMSEA, GFI, AGFI, ECVI, NCP, and SNCP; (ii) value-added fitness statistics, including NFI, RFI, IFI, CFI, and TLI; and (iii) parsimonious fitness statistics, including AIC, CAIC, PNFI, PGFI, and other metrics for model validation.

### 2.5 Quality control

Quality control was conducted for the motor ability assessment and the parent proxy version of the Children’s Motor Health Questionnaire. The testing team was trained on the standardization of the Smart Body Test before conducting the motor ability assessment, and each test was completed by the same group of testers. The motor ability assessment was conducted in accordance with the Smart Body Test workbook combined with an instructional video. The entire testing process was videotaped, and any doubts regarding the on-site assessment were viewed and commented upon by experts to ensure the accuracy and consistency of the assessment. The Parent Proxy Version of the Children’s Exercise Health Questionnaire to be completed via Web WeChat^®^, and the motor ability assessment report could only be accessed after the questionnaire was filled out by the parents, who were informed that the questionnaire tested the child’s physical and mental health and other important content, which was closely linked to the exercise ability assessment to ensure the quantity and quality of the completed questionnaire.

The reliability of the four scales was tested by Cronbach’s alpha coefficient, and the validity was tested by the Kaiser–Meyer–Olkin (KMO) test and Bartlett’s test of sphericity. The Cronbach’s alpha values exceeded 0.7, and KMO values were greater than 0.8 for all four scales, both of which were statistically significant (*p*-value = <0.001), demonstrating that the four scales had good measurement reliability and strong validity.

## 3 Results

### 3.1 Preliminary data analysis for model construction

Table 1 presents the descriptive statistics of the measures used in this study. Before proceeding with further analysis, we assessed the distributional properties of the variables. The skewness and kurtosis indices were within acceptable limits, with skewness coefficients below 2, and kurtosis values below 5 for all indicators, confirming that all indicators were normally distributed. We removed one or two outliers in some measures to minimize their impact on the results.

**TABLE 1:** Descriptive statistics.

Measure	Mean	SD	Min	Max	Skewness	Kurtosis
A: MA	89.61	0.37	64.06	99.55	−0.89	0.15
A1: speed	92.71	0.47	55	100	−1.37	1.18
A2: strength	79.71	0.49	50	100	−0.54	−0.20
A3: CA	92.55	0.59	30	100	−1.92	4.25
A4: flexibility	87.26	0.76	50	100	−1.08	−0.12
A5: balance	92.78	0.57	30	100	−1.69	2.73
B: PH	70.03	0.42	39.73	97.95	−0.26	0.98
B1: body shape	78.36	0.73	25	100	−0.99	0.71
B2: PP	64.24	0.51	31.3	100	0.44	1.39
C: MH	47.50	0.33	26.42	85.81	0.54	2.35
C1: EH	63.25	0.53	25	100	−0.44	1.03
C2: self-esteem	47.96	0.70	6.3	100	−0.40	0.39
C3: families	49.69	0.81	12.5	100	0.84	0.42
C4: SC	49.02	0.67	12.5	93.8	−0.12	−0.01
C5: schools	59.77	0.78	12.5	93.8	−0.55	−0.20
D: SEC	47.47	0.39	28.54	74.06	0.55	0.18
D1: SO	43.64	0.67	11.1	86.1	0.24	−0.38
D2: RS	48.79	0.53	11.1	80.6	0.28	0.42
D3: DM	47.47	0.57	0	84.4	−0.29	1.08
D4: SA	49.51	0.45	0	78.6	0.25	1.90
D5: SM	49.59	0.46	27.8	80.6	0.06	0.07
E: MHE	55.62	0.44	29.33	76.31	−0.36	−0.16
E1: SP	47.36	0.80	16.7	91.7	−0.05	−0.51
E2: CE	54.80	0.86	0	100	−0.10	0.05
E3: SI	59.07	0.84	12.5	100	−0.79	−0.12
E4: SE	60.19	0.26	25	75	0.22	4.33
E5: FE	55.63	0.67	18.8	87.5	−0.16	−0.40
F: MHB	48.14	0.52	19.3	73.51	−0.21	−0.31
F1: MB	47.89	0.80	12.5	95.8	0.06	−0.18
F2: lifestyle	43.60	0.77	15	80	−0.13	−0.85
F3: PB	52.09	0.46	20	75	−0.28	1.02
F4: adaptation	49.62	0.57	18.8	81.3	−0.07	−0.09

Min, minimum; Max, maximum; MA, motor ability; PH, physical health; MH, for mental health; SEC, social and emotional competencies; MHE, motor health environment; MHB, motor health behavior; CA, coordination ability; PP, physiological perception; EH, emotional health; SC, social contacts; SO, social awareness; RS, relationship skills; DM, responsible decision-making; SA, self-awareness; SM, self-management; SP, sports policies; CE, community environment; SI, sports institutions; SE, school environment; FE, family environment; MB, motor behavior; PB, psychological behavior.

Table 2 presents the correlations between these measures. In general, the total motor health scores of preschoolers aged 5–6 years showed highly significant positive correlations (*p*-value < 0.01) at the 0.01 level (two-tailed) with motor ability, physical health, mental health, social and emotional competence, motor health environment, and motor health behavior. Among these relationships, motor ability had a weak negative correlation with physical health (*r* = −0.039, *p*-value > 0.05); a highly significant positive correlation with mental health at the 0.01 level (two-tailed) (*r* = 0.150, *p*-value = 0.002 < 0.01); and a highly significant positive correlation with social and emotional competence at the 0.01 level (two-tailed) (*r* = 0.281, *p*-value = 0.000 < 0.001); a low strength significant negative correlation with motor health environment at the 0.01 level (two-tailed) (*r* = −0.392, *p*-value = 0.000 < 0.001); and a significant positive correlation with motor health behavior at the 0.01 level (two-tailed) (*r* = 0.284, *p*-value = 0.000 < 0.001).

**TABLE 2:** Correlation between all study variables.

Measure	1	2	3	4	5	6	7	8	9	10	11	12	13	14	15	16
MA	–															
Speed	0.601**	–														
Strength	0.555**	0.366**	–													
CA	0.769**	0.290**	0.183**	–												
Flexibility	0.604**	0.201**	0.216**	0.344**	–											
Balance	0.620**	0.190**	0.233**	0.320**	0.224**	–										
PH	−0.039	0.003	0.067	−0.049	−0.015	−0.096	–									
Body shape	−0.220**	−0.071	−0.076	−0.171**	−0.164**	−0.195**	0.699**	–								
PP	0.165**	0.075	0.170**	0.102*	0.142**	0.060	0.701**	−0.020	–							
MH	0.150**	0.043	0.214**	0.106*	0.059	0.072	0.172**	−0.074	0.314**	–						
EH	0.137**	0.053	0.148**	0.098*	0.076	0.072	0.215**	−0.087	0.387**	0.494**	–					
Self-esteem	0.046	0.015	0.100*	0.037	−0.016	0.017	0.028	−0.064	0.104*	0.713**	0.029	–				
Families	0.528**	0.274**	0.433**	0.361**	0.332**	0.309**	0.117*	−0.154**	0.317**	0.673**	0.285**	0.288**	–			
SC	−0.343**	−0.186**	−0.189**	−0.214**	−0.275**	−0.247**	0.065	0.119*	−0.028	0.136**	−0.004	0.144**	−0.189**	–		
Schools	−0.509**	−0.330**	−0.302**	−0.352**	−0.337**	−0.312**	0.054	0.178**	−0.102*	0.266**	−0.017	0.070	−0.274**	0.308**	–	
SEC	0.281**	0.143**	0.308**	0.155**	0.166**	0.170**	0.000	−0.094	0.094	0.319**	−0.131**	0.337**	0.437**	−0.051	−0.121*	–
SO	0.090	0.074	0.194**	0.001	0.087	−0.004	0.075	−0.043	0.148**	0.266**	0.086	0.183**	0.281**	0.001	−0.023	0.681**
**Measure**	**17**	**18**	**19**	**20**	**21**	**22**	**23**	**24**	**25**	**26**	**27**	**28**	**29**	**30**	**31**	**32**
RS	0.311**	–														
DM	0.177**	0.415**	–													
SA	0.285**	0.476**	0.562**	–												
SM	0.207**	0.474**	0.534**	0.670**	–											
MHE	0.030	0.122*	0.100*	0.255**	0.329**	–										
SP	0.111*	0.217**	0.089	0.334**	0.430**	0.703**	–									
CE	0.019	0.025	0.021	0.055	0.131**	0.687**	0.357**	–								
SI	0.009	0.028	−0.065	0.161**	0.175**	0.736**	0.491**	0.391**	–							
SE	0.016	0.170**	0.140**	0.196**	0.250**	0.499**	0.369**	0.199**	0.247**	–						
FE	−0.045	0.033	0.184**	0.127*	0.138**	0.542**	0.102*	0.130**	0.141**	0.253**	–					
MHB	0.261**	0.464**	0.539**	0.573**	0.615**	0.239**	0.242**	0.071	0.062	0.261**	0.214**	–				
MB	0.222**	0.311**	0.358**	0.416**	0.401**	0.236**	0.181**	0.124*	0.075	0.207**	0.215**	0.816**	–			
Lifestyle	0.210**	0.429**	0.487**	0.529**	0.636**	0.228**	0.296**	0.047	0.097	0.248**	0.137**	0.820**	0.476**	–		
PB	0.107*	0.363**	0.397**	0.422**	0.462**	0.211**	0.214**	0.046	0.077	0.297**	0.163**	0.744**	0.482**	0.571**	–	
Adaptation	0.238**	0.349**	0.446**	0.401**	0.396**	0.026	0.029	−0.038	−0.085	0.064	0.136**	0.693**	0.395**	0.443**	0.437*	–

1, MA; 2, speed; 3, strength; 4, CA; 5, flexibility; 6, balance; 7, PH; 8, body shape; 9, PP; 10, MH; 11, EH; 12, self-esteem; 13, families; 14, SC; 15, schools; 16, SEC; 17, SO; 18, RS; 19, DM; 20, SA; 21, SM; 22, MHE; 23, SP; 24, CE; 25, SI; 26, SE; 27, FE; 28, MHB; 29, MB; 30, lifestyle; 31, PB; 32, adaptation. *, Significantly correlated at the 0.05 level (two-tailed); **, Significantly correlated at the 0.01 level (two-tailed).

Physical health had a weak but significantly positive correlation with mental health (*r* = 0.172, *p* = 0.000 < 0.001), was completely uncorrelated with social and emotional competence (*r* = 0.000), weak negative correlation with motor health environments (*r* = −0.075, *p*-value > 0.05); and weak negative correlation with motor health behaviors (*r* = −0.039, *p*-value > 0.05). Mental health had a highly significant positive correlation with social and emotional competence at the 0.01 level (two-tailed) (*r* = 0.319, *p*-value = 0.000 < 0.001); a weak significant negative correlation with motor health environment at the 0.05 level (two-tailed) (*r* = −0.105, *p*-value = 0.034 < 0.05); and a weak negative correlation with motor health (*r* = 0.170, *p*-value = 0.001 < 0.01). Social-emotional competence had a highly significant positive correlation with motor health environment at the 0.01 level (two-tailed) (*r* = 0.192, *p*-value = 0.000 < 0.001) and with motor health behavior at the 0.01 level (two-tailed) (*r* = 0.637, *p*-value = 0.000 < 0.001). There was a highly significant positive correlation (*r* = 0.239, *p*-value = 0.000 < 0.001) at the 0.01 level (two-tailed) between the motor health environment and motor health behavior.

### 3.2 Constructing an evaluation model for preschool children

**FIGURE 1:**
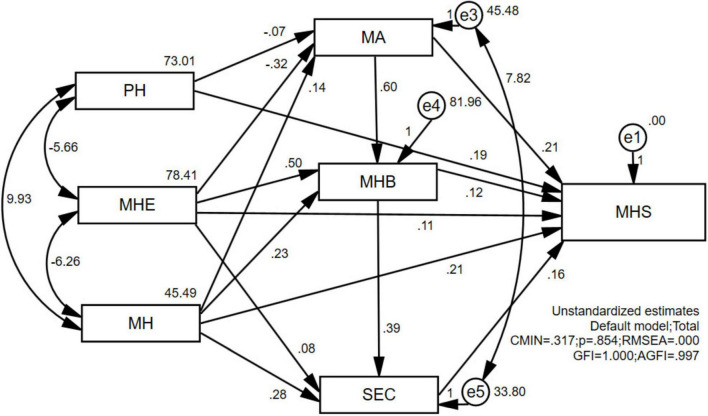
Unstandardized parameter estimation model graph.

**FIGURE 2:**
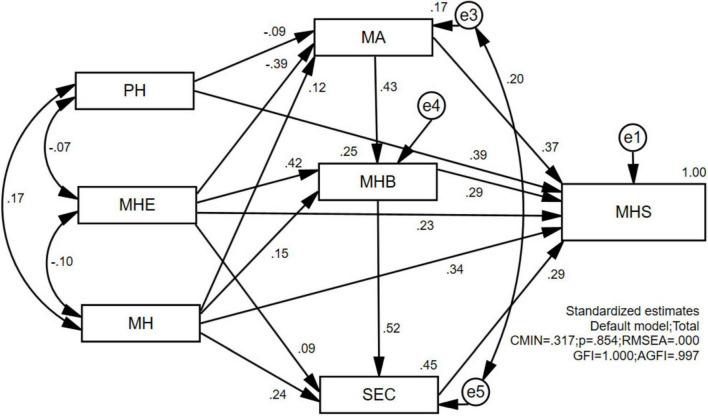
Standardized parameter estimation model graph.

IBM^®^ SPSS^®^ Amos™ 24.0 was used to statistically analyze the data and develop a structural equation model for children’s sports health. In this study, the data of 410 preschoolers were obtained by means of pre-assessment and questionnaires. Both the unstandardized parameter assessment model (Figure 1) and the standardized parameter assessment model (Figure 2) were constructed by estimating the regression coefficients using the method of great likelihood in accordance with the key steps of model conceptualization: construction of the path diagram, model validation, model recognition, parameter estimation, assessment of the fitness of the model, modification of the model, and model revision and calibration.

In the path analysis in Figure 1, the numbers on the double arrows of the path analysis represent the covariance between the two variables, and the numbers next to the observed variables and error terms are the variances, all of which are positive. If a variance value is negative, it suggests that the model estimation has unreasonable parameters. The path coefficient of Physical Health (PH) → Motor Ability (MA) is −0.07, and the path coefficient of Motor Health Environment (MHE) → MA is −0.32, which indicates that the effect between the variables is negative, while all other path coefficients are positive.

In Figure 2, the numbers on the double arrows in the path analysis represent the correlation coefficients between the two variables, while the numbers on the single arrows indicate the path coefficients (standardized regression coefficients) from exogenous to endogenous variables. The absolute values of the path coefficients are all less than 1, while the absolute values of the correlation coefficients are greater than 1, indicating the existence of irrational parameters in the model. In terms of standardized regression coefficients, the path coefficient of PH → MA is −0.09, the path coefficient of MHE → MA is −0.39, and the other path coefficients are positive. The coefficients are all positive. The three variables of MHE, Mental Health (MH) and MA can explain 25% of the variance of the variable of Motor Health Behavior (MHB) of preschool children, and the path coefficient of MHE, MH, and MA is −0.09, and all other path coefficients are positive. The variables MHE, MH, and MHB explained 45% of the variance in the variable Social and Emotional Competence (SEC) in preschoolers.

### 3.3 Structural model variable relationships

The effect values of the variables in the path analysis structural model are listed in Table 3. The direct effect value of MA on Motor Health Score (MHS) was 0.212, the indirect effect value was 0.107, and the total effect value was 0.319, which is statistically significant (*p*-value = 0.000 < 0.001). The direct effect value of MH on MHS was 0.213, the indirect effect value was 0.129 and the total effect value was 0.342, which is statistically significant (*p*-value = 0.000 < 0.001). The direct effect value of MHB on Total MHS was 0.118, the indirect effect value was 0.061, and the total effect value was 0.179, which was statistically significant (*p*-value = 0.000 < 0.001). The direct effect value of MHE on Total MHS was 0.109, the indirect effect value was −0.001, and the total effect value was 0.108, which was statistically significant (*p*-value = 0.000 < 0.001). The direct effect value of SEC on Total MHS was 0.156, the indirect effect value was 0.000, and the total effect value was 0.156, which was statistically significant (*p*-value = 0.000 < 0.001). The direct effect value of PH on Total MHS was 0.191, the indirect effect value was −0.024, and the total effect value was 0.167, which was statistically significant (*p*-value = 0.000 < 0.001).

**TABLE 3:** Structural model variable relationships.

Variable relations	Direct effects	Indirect effects	Total effects (*P*)
MA → MHB	0.601	–	0.601 (0.000)***
MA → SEC	–	0.234	0.234 (0.000)***
MA → MHS	0.212	0.107	0.319 (0.000)***
MH → MA	0.137	–	0.137 (0.007)**
MH → MHB	0.232	0.082	0.314 (0.000)***
MH → SEC	0.281	0.122	0.404 (0.000)***
MH → MHS	0.213	0.129	0.342 (0.000)***
MHB → SEC	0.389	–	0.389 (0.000)***
MHB → MHS	0.118	0.061	0.179 (0.000)***
MHE → MA	−0.323	–	−0.323 (0.000)***
MHE → MHB	0.497	−0.194	0.303 (0.000)***
MHE → SEC	0.084	0.118	0.202 (0.014)**
MHE → MHS	0.109	−0.001	0.108 (0.000)***
SEC → MHS	0.156	–	0.156 (0.000)***
PH → MA	−0.074	–	−0.074 (0.056)
PH → MHB	–	−0.045	−0.045
PH → SEC	–	−0.017	−0.017
PH → MHS	0.191	−0.024	0.167 (0.000)***

MA, motor ability; PH, physical health; MH, for mental health; SEC, social and emotional competencies; MHE, motor health environment; MHB, motor health behavior; MHS, total motor health score. *p < 0.05; **p < 0.01; ***p < 0.001.

### 3.4 Statistical test of model fitness

Looking at the validation through the overall structural equation model fitness evaluation, the results of the validation are shown in Table 4. In terms of the absolute fitness indicators, the degree of freedom of the theoretical model is equal to 2, the Chi-square value of the overall fitness = 0.317, the probability of significance (*p*-value = 0.158 > 0.05), the RMSEA value = 0.000 < 0.05, the GFI value is equal to 1.000 > 0.90, the AGFI value = 0.997 > 0.90, the null hypothesis is accepted. From the value-added fitness indicators, NFI value = 1.000 > 0.90, IFI value = 1.000 > 0.90, TLI value = 1.003 > 0.90, and CFI value = 1.000 > 0.90, which all meet the criteria of model acceptability. Based on the parsimony fitness index, the Chi-square degrees of freedom ratio is 0.158 < 2.000, indicating a good model fit. The AIC value (52.317) and ECVI value (0.128) of the theoretical model are both lower than those of the independent model and saturated models. The CN value is 7,742 at the α = 0.05 significance level, and 11,900 at the α = 0.01 level, confirming that the model meets the fitness criteria.

**TABLE 4:** Default model fitness validation results.

Model	χ^2^	*df*	χ^2^*/df*	*P*	RMSEA	NFI	IFI	CFI	GFI	AIC	ECVI
Default	0.317	2	0.158	0.854	0.000	1.000	1.000	1.000	1.000	52.317	0.128
Saturated	0.000	0				1.000	1.000	1.000	1.000	56.000	0.137
Independence	6388.608	21	304.219	0.000	0.861	0.000	0.000	0.000	0.555	6,402.608	15.654

χ^2^ statistic for all models statistically significant at *p* < 0.001. df, degrees of freedom; χ^2^*/df*, chi-square ratio of degrees of freedom; RMSEA, root mean square error of approximation; NFI, normed fit index; IFI, incremental fit index; CFI, comparative fit index; GFI, goodness-of-fit index; AIC, Akaike information criterion; ECVI, expected cross-validation index.

Overall, in terms of the main fitness statistics, the theoretical causal model of preschool children’s sports health aligns well with the actual data. In addition, the correction indicators and desired parameter change table show that covariance settings, variance settings, and incremental path coefficients are not available, which indicates that there is no need to release additional parameters in the model.

The multi-cluster model test can further validate whether the preset model can be recognized properly and whether there is any contradiction between the overall test results of the preset model and the measurement model with more restrictive parameters. Therefore, this study implemented a multi-cluster structural equation model fitness evaluation. The validation results are presented in Table 5. In terms of absolute fitness indicators, divided into five cluster models according to different genders and different regions, the Chi-square values of fitness were 16.691, 3.207, 3.511, 4.407, and 4.227, and the probability values of significance were *p*-value = 0.544, 1.000, 0.743, 0.622, and 0.646, which were all greater than 0.05. The five cluster models RMSEA = 0.000 < 0.05, and GFI values equal to 0.989, 0.998, 0.998, 0.997, 0.997, and 0.997, all greater than 0.90, and the null hypothesis was accepted. In terms of value-added fitness indicators, NFI values are equal to 0.997, 1.000, 0.999, 0.999, 0.999, and 0.999, respectively, all greater than 0.90; IFI values are equal to 1.000, 1.002, 1.000, 1.000, and 1.000, all greater than 0.90; the five cluster models have CFI values of 1.000 > 0.90, and all value-added fitness indicators meet the acceptable standard of the model. From the parsimonious fitness indicators, the Chi-square degrees of freedom ratios were 0.927, 0.178, 0.585, 0.735, and 0.704, all less than 2.000, and the AIC and ECVI values of the preset models were smaller than the independent model values and, at the same time, smaller than the saturation model values, so that the models could reach the standard of parsimonious fitness.

**TABLE 5:** Multi-cluster model fitness validation results.

Default model	*N*	χ^2^	*df*	χ^2^*/df*	*P*	RMSEA	NFI	IFI	CFI	GFI	AIC	ECVI
**Gender group**												
Total	410	0.317	2	0.158	0.854	0.000	1.000	1.000	1.000	1.000	52.317	0.128
Male	201	16.691	18	0.927	0.544	0.000	0.997	1.000	1.000	0.989	92.691	0.227
Female	209	3.207	18	0.178	1.000	0.000	1.000	1.002	1.000	0.998	79.207	0.190
**Regional group**												
Region 1	143	3.511	6	0.585	0.743	0.000	0.999	1.000	1.000	0.998	159.511	0.392
Region 2	150	4.407	6	0.735	0.622	0.000	0.999	1.000	1.000	0.997	160.407	0.387
Region 3	117	4.227	6	0.704	0.646	0.000	0.999	1.000	1.000	0.997	160.227	0.421

χ^2^ statistic for all models statistically significant at *p* < .001. *df*, degrees of freedom; χ^2^*/df*, chi-square ratio of degrees of freedom; RMSEA, root mean square error of approximation; NFI, normed fit index; IFI, incremental fit index; CFI, comparative fit index; GFI, goodness-of-fit index; AIC, Akaike information criterion; ECVI, expected cross-validation index.

The model fitness statistics for multiple clusters indicate that the theoretical model diagram of preschool children’s sports health aligns well with the actual data across multiple clusters. The covariance settings, variance settings, and incremental path coefficients are not shown in the table of correction indices and desired parameter changes, indicating that there is no need to modify the parameters of the model and that the model is stable. Overall, the hypothetical models for both gender and regional clusters can be adapted to the sample data, and the single covariance structure relationship of a single sample has an equivalent relationship with several parallel covariance structures. This shows that the theoretical model is highly stable, adaptable, and broadly applicable.

## 4 Discussion

Structural equation modeling has traditionally been applied to theoretical testing and allows for optimal predictive analysis through SEM (Evermann and Tate, 2016). Model evaluation is a complex process of combining statistical criteria with philosophical, historical, and theoretical elements, attempting to reconcile objective and subjective criteria (Bagozzi and Yi, 1988). In this study, SEM was used to examine relationships between evaluation indicator variables, and interactions between variables in terms of both their intensity of influence and throughput coefficients (Yang et al., 2023).

Preschool children have the highest rate of learning and cognitive development, and exercise is beneficial for their physical and MH development (Jia et al., 2021). Furthermore, motor skill development and physical education classes have a significant impact on preschoolers’ physical and mental health, and on academic learning (Wang et al., 2023). Promoting socio-emotional competence is a key factor in academic success and positive behavioral outcomes for children aged 3–6 (Martinsone, 2016). Emotional competence also has long-term effects on social competence in the early preschool years (Denham and Bassett, 2019). Promoting physical activity in preschoolers is a critical intervention to optimize health and prevent chronic disease. It is crucial to establish and maintain a healthy and active lifestyle among preschoolers, with parents being the most powerful role models and who control young children’s behaviors, diets, and activity environments (Goldfield et al., 2012). One study assessed the best and worst physical activity (PA) practices in two Portuguese kindergartens, with motor and socio-emotional competence assessed through standardized motor skill tasks and parental reports of children’s behavior. Children in the kindergarten with physical activity best practices demonstrated significantly better motor skills, and no statistically significant differences were found in socio-emotional competence scores (Moreira et al., 2023). One study found that a Positive Action preschooler program improved social-emotional competence and health behaviors in low-income children (Schmitt et al., 2018). Physically oriented interventions in an educational context are effective in improving specific socio-emotional competencies in preschool children (Dias Rodrigues et al., 2022). Early childhood education is the new focus of European education trends and is a means to improve the quality of life and outdoor activities in any season and weather, contributing to the development of social and emotional competencies for preschool children (Clipa and Cîmpan, 2020). Emotional competence is a key set of skills for success in life, and teachers can facilitate its development among preschoolers (Denham and Bassett, 2019). Motor behavior development contributes to basic motor skills, and promoting appropriate motor behavior is essential for children’s health (Kracht et al., 2020). Shaping and promoting positive behaviors early in life is critical to developing lifelong healthy habits, and a child’s environment has a powerful influence on behavior (Adamo et al., 2014).

After an in-depth exploration, this study constructed a preschool children’s motor health model, revealing the internal relationship between preschool children’s motor health evaluation indicators. Several studies have identified factors that affect sports health (Bremner et al., 2020; Hamer et al., 2012). The fitness indicators of the preset theoretical model reached an acceptable standard, and the null hypothesis was accepted, as seen through the overall structural equation model fitness evaluation verification. Through the multi-cluster SEM fitness evaluation test, the model was tested across five clusters: male, female, region 1, region 2, and region 3. The fitness indices for all clusters met the acceptable standard, and the null hypothesis was accepted, confirming the five clusters model achieved a satisfactory level of fit.

The total motor health scores of Chinese preschool children aged 3–6 years showed highly significant positive correlations with motor ability, physical health, mental health, social and emotional competence, motor health environment, and motor health behavior. In the path analysis structural model, the total effect sizes of the effects on motor health, in order of magnitude, were as follows: MH (0.342), MA (0.319), MHB (0.179), PH (0.167), SEC (0.156), and MHE (0.108), all of which were statistically significant (*p*-value = 0.000 < 0.001). However, our study revealed a path coefficient of MHE → MA at –0.39, which contradicts previous research emphasizing the positive effects of motor health environments on motor abilities (Christian et al., 2022; Ng et al., 2020; Grande et al., 2016). This discrepancy may originate from cross-cultural and cross-regional sampling differences, highlighting that the “geographical” factor exhibits significant regional heterogeneity in shaping Chinese preschoolers’ motor development. Notably, the motor health environment demonstrates bidirectional impacts (β = –0.39), potentially enhancing or inhibiting motor competencies contingent upon contextual specificities. This finding is important for understanding the motor health model in preschoolers and provides new contributions and ideas for future research. In the course of the study, we were deeply impressed by the challenges of constructing a model for evaluating preschool children’s motor health based on the multidisciplinary intersection of motor science, physical health, mental health, ecological environment, social learning, comprehensive evaluation, mathematical modeling, mathematical statistics, and social and emotional competence. However, we eventually overcame these difficulties through our unremitting efforts.

The study of preschool children’s motor health levels through mathematical modeling was conducted to better explore the field and introduce new research tools, techniques, and methods to improve the accuracy and reliability of the study. We also established the SEM based on Du and Choosakul (2025), Ayaz-Alkaya and Kulakçı-Altıntaş (2021), and Niemistö et al. (2020). Therefore, this study selected the key indicators of motor ability, physical health, mental health, motor health behavior, social and emotional competence, and motor health environment, to evaluate the level of preschool children’s motor health based on their intrinsic connections. These factors were closely integrated with the practice of motor health work, and underwent appropriate processing and refining. Additionally, statistical methods and mathematical modeling were applied to develop a structured evaluation model, enabling an objective evaluation of different evaluation categories, and their advantages and disadvantages, to provide a basis for sports health decision-making.

Through an in-depth discussion of the theoretical foundation and the factors influencing motor health, after a series of rigorous analysis and argumentation, this research is of great significance for understanding the intrinsic principles of preschool children’s motor health. It also introduces a new way of thinking and a practical methodology for the evaluating the motor health of preschool children. This study evaluated preschool children’s motor health level based on cross-disciplinary theories such as Bronfenbrenner’s ecosystem, Bandura’s social learning, CASEL’s social and emotional learning, comprehensive evaluation model, exercise science, and physical and mental health principles, An evaluation model was constructed, including motor ability, physical health, mental health, motor health behavior, asocial and emotional ability, and motor health environment. Based on their intrinsic connections, we processed and refined them and applied them to the practice of motor health work. We used the evaluation model to seek a more objective judgment on the categories of the evaluation objects and their advantages and disadvantages, and to provide a decision-making framework for the improvement of the motor health level of preschool children.

### 4.1 Strengths and limitations

The advantages of this study lie in the successful construction of evaluation model, its practical applications and the rigorous quality control of the data collection. Firstly, based on interdisciplinary theory, the findings reveal the coupling effect of various factors on preschool children’s sports health level. Second, the research results provide guidance to help kindergarten teachers and parents implement scientific sports and health education. Finally, the quality control was mainly aimed at the evaluation of preschool children’s sports ability and the administration of the parents’ proxy version of sports health, ensuring the quality level of the research.

This study has some limitations. When selecting the research sample, only five kindergarten preschools in Guizhou Province, a western region of China, were chosen as the research subjects. The adaptive range and stability of SEM needs to be further verified and adapted in future research. Future research plans will expand the sampling to encompass multiple regions, with explicit emphasis on cross-cultural applicability and broader demographic representation, thereby reinforcing the model’s global relevance. An additional limitation of this study is the potential bias inherent in parent-reported data and measurement error associated with cluster sampling methodology. We believe that there is still much space for the development of statistical modeling for preschool children’s motor health, and future research can further expand the breadth and depth in terms of geography, age, culture, ethnicity, and country on the basis of the present study. To summarize, this study has contributed to the development of motor health assessment models for preschool children; however, there are still many issues that deserve further exploration. We look forward to more scholars joining this research field to promote the progress and development of preschool children’s motor health. We hope that the results of this study will provide useful references and insights for future research.

## 5 Conclusion

The Chinese preschool children’s motor health evaluation model successfully passed the fitness index test, indicating that the theoretical model is highly stable, adaptable, and widely acceptable. This study challenged the multidisciplinary cross-construction of a preschool children’s motor health evaluation model, which contributed to the development of preschool children’s motor health levels and provided useful references and inspiration for subsequent studies.

## Data Availability

The information, data, figures, tables, and raw data supporting the conclusions of this article will be made available by the authors, without undue reservation. For further inquiries, please contact the corresponding author at 726303420@qq.com.
